# Step-by-step loupes-mTESE in non-obstructive azoospermic men, a retrospective study

**DOI:** 10.1186/s12610-019-0091-9

**Published:** 2019-07-15

**Authors:** Amin Bouker, Lazhar Halouani, Mahmoud Kharouf, Habib Latrous, Mounir Makni, Ouafi Marrakchi, Raoudha Zouari, Salima Fourati

**Affiliations:** CPSR, department of AMP, Clinique Les Jasmins, Tunis, Tunisia

**Keywords:** × 6 loupes, Micro TESE, Step-by-step, Non-obstructive azoospermia, Infertility, Andrological surgery, ICSI, Loupes × 6, Microdissection, Azoospermie non obstructive, Infécondité, Chirurgie andrologique, ICSI

## Abstract

**Background:**

Men with non-obstructive azoospermia (NOA) may have sperm in their testes and a procedure of sperm retrieval and assisted reproduction is required in them to allow fertility. Standard procedures such as fine needle aspiration (FNA) and conventional testicular sperm extraction (cTESE) harvest random samples with a sperm retrieval rate (SRR) of 45%. Microdissection testicular sperm extraction (mTESE) is nowadays considered to be the most accurate technique to retrieve sperm in men with NOA. This procedure can identify dilated tubules that are more likely to contain viable sperm with a SRR of 60%.

**Results:**

In our center, testicular biopsy was conducted in a standard fashion in 321 patients with NOA until March 2003. From then to December 2017, due to the lack of an operating microscope, we used 6 fold magnifying loupes to perform a step-by-step macro- mTESE in 1050 patients. Sperm was found in the first testis in 61% of the cases, leading to stop the procedure with less testicular damage. We increased our SRR from 43 to 51.8% in an acceptable operating time of 75mn for both sides.

**Conclusions:**

In institutions where surgeons cannot afford an operating microscope, this modified mTESE technique using × 6 magnifying loupes is reliable, especially in patients with low testicular volumes and high FSH, in whom dilated tubules can be easily identified from the surrounding tissue.

## Résumé

**Contexte:**

Les patients ayant une azoospermie non obstructive confirmée peuvent néanmoins présenter des spermatozoïdes intratesticulaires nécessitant un prélèvement chirurgical en vue d’une injection intra cytoplasmique d’un spermatozoïde (ICSI). L’aspiration à l’aiguille ainsi que la biopsie classique à ciel ouvert ne permettent qu’un prélèvement aléatoire à l’aveugle assorti d’un taux de positivité de 45%. La biopsie avec microdissection sous microscope est. désormais considérée comme le « gold standard » et permet d’identifier les foyers de tubes séminifères dilatés qui sont le plus à même de contenir des spermatozoïdes mobiles.

**Résultats:**

Dans notre centre d’Assistance Médicale à la Procréation (AMP), jusqu’en février 2003, le recueil de spermatozoïdes pour ICSI a été réalisé par une biopsie classique chez 321 patients avec une positivité de 43%. De mars 2003 à décembre 2017, du fait de l’absence de microscope opératoire, nous avons adapté le prélèvement microchirurgical à des loupes de fort grossissement (× 6) et pratiqué cette technique simplifiée chez 1050 patients. Les fragments sont examinés en extemporané par les embryologistes et chez 61% des patients, la positivité de la biopsie dans le premier testicule prélevé permet de sursoir à l’exploration du côté controlatéral, évitant ainsi une dissection inutile et potentiellement délétère. Grâce à cette modification, nous sommes passés de 43% à 51,8% de positivité avec un temps opératoire moyen de 75mn pour les 2 côtés.

**Conclusion:**

Dans les centres d’AMP où l’on ne dispose pas de microscope opératoire ou lorsque le programme ne permet pas d’allouer une longue durée opératoire à la biopsie testiculaire sans compromettre le reste de l’activité chirurgicale, l’utilisation de loupes à fort grossissement (× 6) permet l’amélioration des résultats de la biopsie, particulièrement chez les patients présentant un petit volume testiculaire et une FSH élevée.

## Introduction

About 10% of infertile men have primary testicular failure with azoospermia [1] in whom non-obstructive azoospermia (NOA) accounts for 70% of the cases [2]. In these patients, testicular biopsy has been considered as a diagnostic tool to assess male fertility although a single biopsy is associated to a poor SRR compared to conventional testicular sperm extraction (cTESE) [3].

In 1997, Schlegel et al. described a new technique, based on the idea that most men with NOA have heterogeneity within their testes and may demonstrate isolate foci of spermatogenesis [4] that can be retrieved microsurgically, using a 25 fold microscope.

In the era of intracytoplasmic sperm injection (ICSI), several studies in the literature supported that microdissection is a more reliable technique to retrieve sperm, with up to 66% SRR [3, 5] compared to cTESE. It also allows reducing the complication rates since intratesticular vessels are easily identified and smaller specimen are retrieved with better quality tissue [6].

This procedure is very time consuming. Ramasamy et al. published that the median operating time was 146mn and even after a four-hour microdissection with no sperm found yet, the probability of a positive biopsy was 37% if the procedure went on [7].

We conducted a retrospective study from March 2003 to December 2017 to assess the utility of × 6 magnifying loupes together with deep Schlegel-like dissection for sperm retrieval in patients with NOA.

## Patients and methods

### Study population

In infertile couples, male partners were evaluated by medical history and physical examination. At least 2 semen analyses were done separated by a 3 month-period. Testicular volume was assessed by untrasound. Follicle-stimulatin-hormone (FSH) and testosterone levels were mandatory. All patients underwent karyotype analyses and search for microdeletions of the Y chromosome.

In our country, due to the lack of coverage of the whole ICSI procedure by the social security and the health insurance, patients usually undergo asynchronous testicular biopsy in order to avoid an unnecessary ovarian stimulation, all the more since we cannot offer donor sperm as a back plan.

Synchronous testicular biopsy was done in case of cryptozoospermia, when no motile sperm were found in the ejaculate the day of surgery. Half of our patients came from abroad, mainly Algeria, Lybia and Mali and their medical files were forwarded to our team prior to testicular biopsy.

All the procedures were performed by a single urologist. All patients were discharged the day of surgery and the gynecologist who was in charge of the female partner was informed of the outcome of the procedure. In our institution, we are palnning to hire a psychologist, not only to inform patients of the negative outcome of the procedure but also to assist them in a decision making process to choose between adoption and sperm donor which is only available abroad.

### Exclusion criteria

In order to evaluate our technique in NOA patients, we had the following exclusion criteria.

1. If the vas deferens seemed thinner than usual at clinical palpation, an ultrasound examination of the whole seminal duct was performed transrectally in order to exclude any patient with partial congenital absence of vas deferens.
2. In some patients, we had the chance to collect former semen analyses showing normal values or at least normal or high concentrations of sperm, indicating that an obstruction may have occured, leading to a secondary infertility. Before albugineal incision, despite the absence of enlarged epididymis at clinical palpation and especially if questioning showed any history of orchitis, we paid special attention to exclude cases where × 6 loupes demonstrated any unpalpable tiny dilated tubule in the caput of the epididymis, showing that an obstruction occured. Sometimes, we even dissected out the efferent ducts and if they contained sperm due to a very proximal obstruction, the patient was excluded.
3. Finally, even if the epididymis appeared to be normal, if the very first superficial biopsies showed multiple areas with dilated seminiferous tubules (ST) containing sperm and if these foci were not separated by ubnormal surrounding tubules, the patient was more likely to have obstruction than hypospermatogenesis and was excluded from the cohort after histological assessment.

Thereby, all the selected patients reported in our study did have pure NOA.

### Classification of testicular pathology

We divide NOA patients into 4 categories according to the current European Association of Urology recommendations [8].

1. Seminiferous tubule hyalinization: Extensive intratubular and peritubular hylalinization or absence of seminiferous tubules.
2. Sertoli-cell-only syndrome (SCO): The tubules have reduced diameter with mature Sertoli cells but no germ cells at all.
3. Maturation arrest (MA): Complete arrest at a particular stage.
4. Hypospermatogenesis (HS): Overall reduction of germ cell elements to a varying degree with all stages of spermatogenesis present up to spermatozoa. It is more common to have mixed than pure patterns on testicular biopsy and the specimen is classified according to the most advanced pattern rather than the predominant one.

### Conventional TESE

Before 2003, TESE was conducted in a standard fashion: Midline scrotal incision, delivery of the testis, 4 random biopsies taken as follows: 0.5 cm incision of the tunica albuginea and tissue extraction from the upper pole, lower pole and 2 from the mid part of the testis.

The same procedure is repeated on the controlateral side with no intra operative sperm assessment and one random specimen from each testis is sent to histopathological analysis to assess spermatogenic patterns and check that there is no intratubular germ cell neoplasia.

### × 6 loupe assisted TESE

From March 2003 to December 2017, TESE performed with loupe assistance (6 fold magnifying loupes, Heine Optotechnik, Germany) under spinal anesthesia. The testis is delivered through a median raphe incision which allows easy access to both sides except when the testis is located in a mid/high scrotal position and deserves a transverse incision on its own.

Since SRR is not that much related to testicular size [9], we usually begin with the smaller testis in order to preserve the better one for further surgery and testosterone production.

#### Step1

A limited 2 to 3 cm transverse incision is made on the tunica albuginea in a vessel sparing fashion. The assistant is irrigating copiously the operating field with a saline solution to allow permanent nice visualization of the tubules and if needed, haemostasis is achieved with bipolar electrocautery. Sometimes, superficial dilated tubules which are more likely to contain sperm are identified at once. They are dissected out with a microforceps and placed in a sterile Petri dish with sperm transport buffer and prossessed to the in vitro fertilisation (IVF) laboratory for immediate assessment. The samples are dissected with microforceps by the embryology team before being examined intraoperatively under high power microscopy and if no sperm are found, the suspension is centrifuged and reexamined carefully. If enough motile sperm are available for ICSI and freezing, the procedure may stop, avoiding unnecessary deeper dissection.

#### Step2

**Fig. 1:**
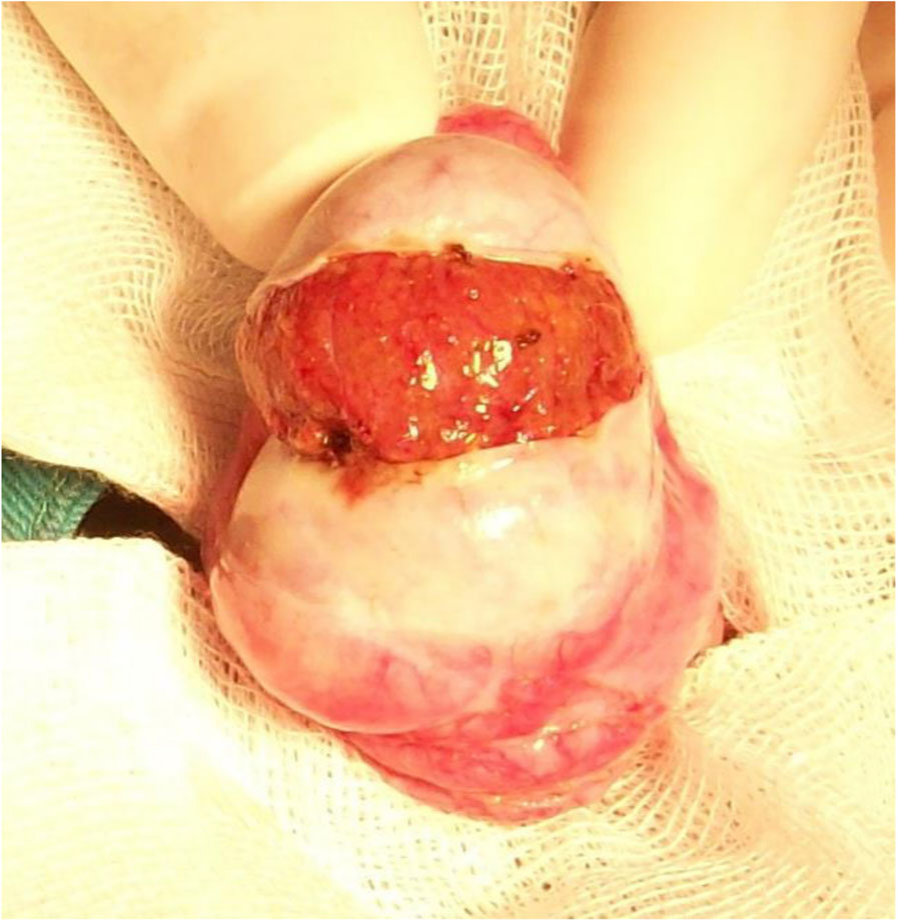
Equatorial incision

The incision is extended equatorially around 180° of the circonference of the testis (Fig. 1) and opening that wide is mandatory to get the testis bivalved smoothly during next step, if necessary.

Superficial testicular tissue is explored and evaluation of size and colour of semineferous tubules is done.

#### Step3

**Fig. 2:**
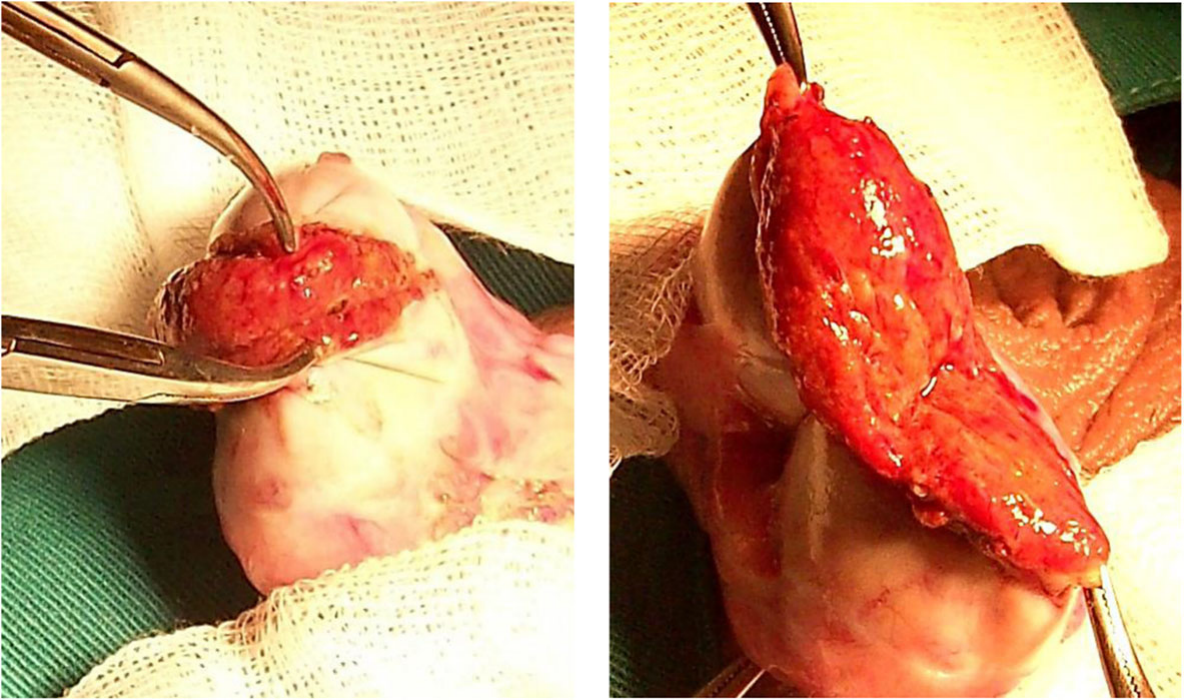
How to bivalve the testis. Legend: From left to right: 1- Mosquito clamps are applied on albugineal edges; 2- The midpart of the testis is splayed out. Note that the clamps are also applied on the parenchyma next to the tunica albuginea.

Two Curved mosquito clamps are applied on each albugineal edge, including the next parenchyma in order to secure the tubules to the tunica albuginea (Fig. 2). This prevents disecting a space between the tunica albuginea and the testicular tissue which may lead to bleeding and haematoma. The surgeon, if right-handed, provides posterior support to the testis with 3 fingers from his left hand, the testis is bivalved under gentle pressure and the deep midpart of the testis is splayed out. The surgeon is exposing the surface of testicular parenchyma with his left thumb and index and dissection goes on with a micro-forceps to identify dilated tubules.

#### Step4

**Fig. 3:**
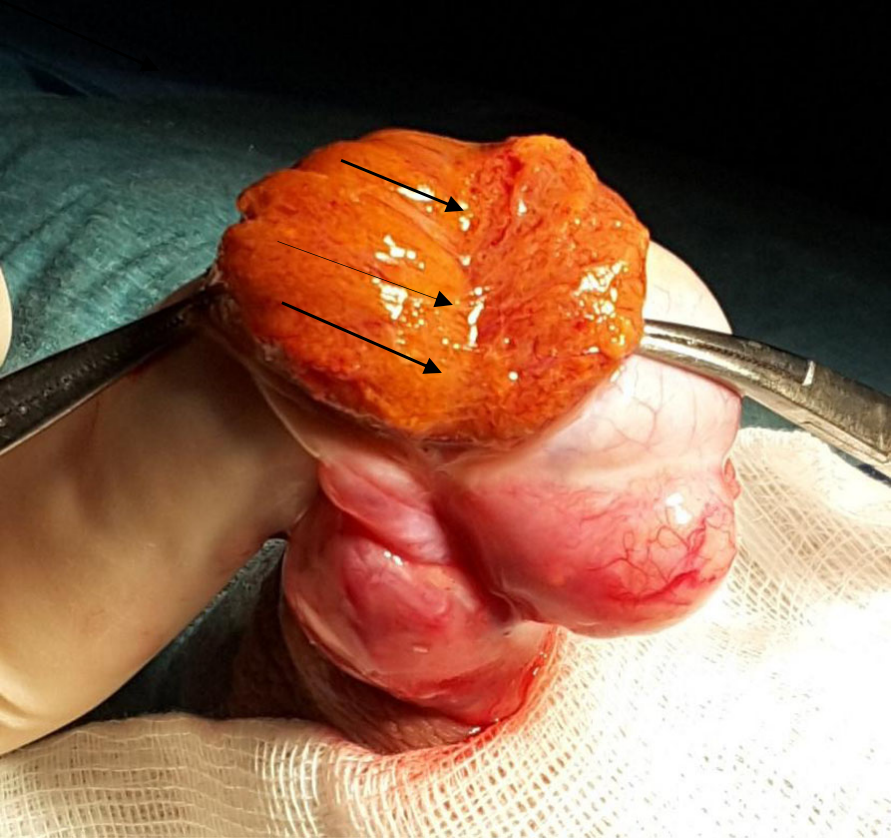
Longitudinal incisions are made at the upper pole

The upper pole is disected out as described by Schlegel [10]: Intra testicular vessels are identified between segments of parenchyma and multiple longitudinal incisions are made between the septae of the testis (Fig. 3), giving access to the deepest areas of the pole where dissection goes on.

#### Step5

The same dissection is performed in the lower pole.

#### Step6

When the entire testis has been harvested and no sperm found yet, dissection of the controlateral side proceeds.

At any step, if dilated tubules are not identified, especially if a uniform pattern cannot allow any size discrimination; several random micro-biopsies are harvested from non fibrotic areas. A 5x5x5 mm testicular specimen with no dilated tubules is taken from each side for histological analysis.

Since our surgical theatre and the embryology laboratory communicate by a pass-through window, we can get initial biological assesment of the specimen within a few minutes.

In Klinefelter [11] and AZFc microdeletion patients [12], we may decide to explore both sides regardless of the intraoperative sperm assessment.

When dissection terminates, the tunica albuginea is closed with a 6/0 non absorbable monofilament. Care must be taken to avoid any underlying vessel injury when suturing. We use either running or interrupted sutures, bringing gently the albugineal edges together without tension in order to avoid any residual intra testicular bleeding turn into a collected haematoma. The tunica vaginalis is closed with a 4/0 Vicryl in a running fashion and the testis is returned to its normal scrotal position. The dartos layer is closed with a 4/0 Vicryl in a running fashion including the entire cut edges for optimal haemostasis. Local anesthesic is infused around the tunica vaginalis, the dartos and the incision site. The skin is closed with a 4/0 Vicryl running suture and a dressing is applied.

A precise operative report is made by the surgeon to specify where dilated tubules have been identified and which segments of the testes were not dissected in order to allow further redo biopsies in these areas.

In some patients, pre operative ultrasound revealed unexpected infra-clinic testicular tumors. Our strategy was to perform tumorectomy with a 5 mm margin of the surrounding tissue and a random biopsy from the remaining parenchyma in order to assess the presence of carcinoma in situ. While intraoperative histopathology assessment was achieved in order to decide if radical orchiectomy should be completed, × 6 loupe TESE opposite to the tumor site was performed as described above.

### Optimisation prior to × 6 loupe assisted TESE

We adviced our patients to stop smoking and lose subsequent weight at least 3 months prior to surgery. None of them was still taking exogenous testosterone within at least 6 months prior to testicular biopsy.

If testosterone level were low, patients were treated with Clomiphene citrate (25 to 50 mg daily, depending on testosterone level), human chorionic gonadotropin (5000 IU twice a week) or a combination of these two medications for at least a 6 month period to optimize endogenous production [10].

In young couples with a palpable varicocele in the male partner, we discussed offering sub inguinal varicocelelectomy with × 6 loupes assistance if the patient was more likely to have hypospermatogenesis or late MA - subnormal testicular volume, normal FSH range, previous biopsy showing late MA -, at least 6 months prior to biopsy [13, 14].

In North Africa, we still have some patients with an undescended testis at the time of biopsy. In such patients, we performed orchiectomy in intraabdominal testes and orchidepexy in other conditions, 6 months prior to testicular biopsy.

### Learning curve

Compared to mTESE, our technique is easier since there is no particular operative dressing to apply to the patient nor specific microsurgical skills required. In the very first cases, due to the magnification of the loupes, even subnormal ST appeared to be dilated and led to failed biopsies. Also, one must avoid small albugineal incisions which may compress the parenchyma that emerges from the testis and show a false aspect of confluent tubules.

**Fig. 4:**
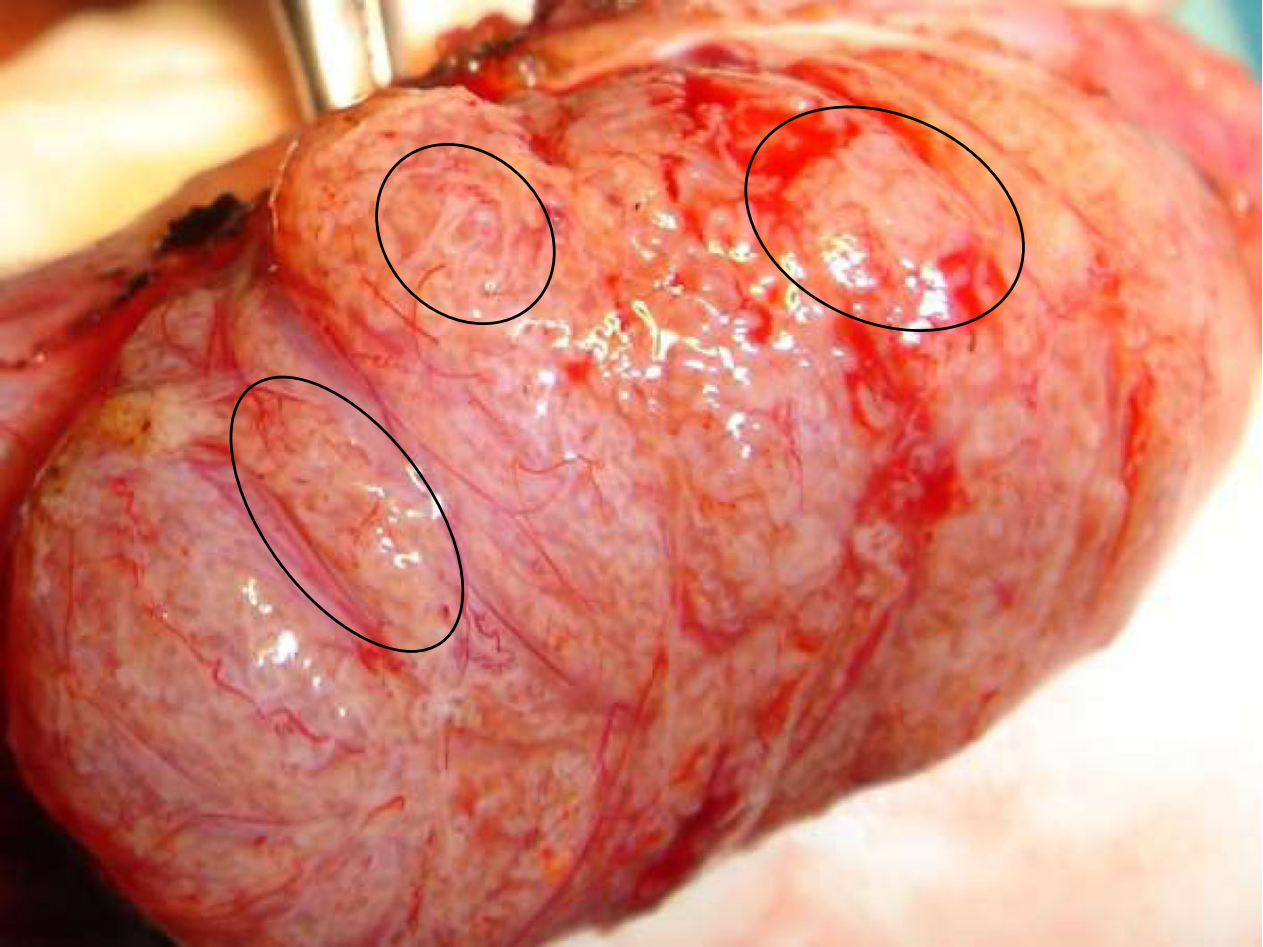
Multiple histological patterns within the testis. Legend: From left to right: 1- Maturation arrest; 2- Sertoli Cell Only pattern; 3- Dilated tubules

It took us a hundred of procedures to establish a nice correlation of 90% between the aspect of the testicular tissue and the intra operative assessment by the embryology team (Fig. 4) that needs its own learning curve. Pictures of the dilated tubules can be taken with a smatphone before they are retrieved. This helps the surgeon to keep in mind the usual ST calibre that is correlated to the presence of sperm. It may happen that a small ST will contain sperm despite its size and on the contrary, dilated tubules may only contain late spermatids. In addition, a better identification of the vessels allowed optimal preservation of blood supply.

## Results

### Sperm retrieval rates

From February 1997 to February 2003, 321 patients had bilateral c-TESE with an overall SRR of 43% (138 patients).

From March 2003 to December 2017, 1050 patients (medium age: 39.1 +/− 6.9) had × 6 loupe-assisted TESE with a SRR of 51.8% (544 patients excluding Klinefelter patients) (Table 1). In our first cases, the mean weight of a biopsy was 10 mg but since 2017, we are harvesting smaller specimen of 1 to 4 mg.

**Table 1:** Comparative sperm retrieval rate between conventional testicular sperm extraction and x6-loupe testicular sperm extraction

Periods	Nb of patients	Technique	SRR (%)
Feb 1997-Feb 2003	321	Bilateral cTESE	43
March 2003-Dec 2017	1050	Loupe TESE	51.8

*SRR* sperm retrieval rate, *cTESE* conventional testicular sperm extraction

Patients older than 35 had better SRR than those under 35 (Table 2).

**Table 2:** Sperm retrieval rate after x6-loupe testicular sperm extraction depending on age

	Age ≤ 35	Age > 35	*p*
Positive biopsy	125	320	0,0006
Failed biopsy	185	292	
SRR (%)	40.3	52.3	

*SRR* sperm retrieval rate, *P* Khi2 test

Viable sperm were identified in the first testis in 332 patients (61%) which led to stop the procedure wheras in 212 patients (39%), contralateral biopsies were needed to find sperm (Table 3). Specimen that showed isolated foci of spermatogenesis were not always located next to the tunica albuginea where cTESE is usually done and in 128 patients (23.5%), only deep areas of the testes contained sperm (Table 3). In 92% of the cases, due to the rapid biological assessment of the specimen, sperm were identified while surgery was going on. Nevertheless, as the surgical procedure was over and no sperm were found in the first testis, 8% of the patients had sperm identified in the very last deepest biopsies of the contralateral side, as the surgeon had left the operating theatre. Standard operative time with loupe assistance was 75mn for both sides (range 10-150mn).

**Table 3:** Location of foci of spermatogenesis

Loupe TESE	First testis	Contralateral testis	Superficial area	Deep area
n	332	212	416	128
SRR (%)	61	39	76.5	23.5

*SRR* sperm retrieval rate

### Post-operative events

We had some postoperative incidents: 4 haematomas after cTESE. One was evacuated on day one postoperatively and 3 were ignored and thus led to testicular atrophy. Since we had loupe assistance which allowed better visualisation of testis vascularity, we had only one patient with clinical haematoma that was followed up and disappereared completely on ultrasound evaluation after a 6 month period. Due to usual uneventfull postoperative recovery, only patients with scrotal pain, swelling or fever came back to our institution for clinical and ultrasound examination, which happened very seldom since we used loupe assistance. Testosterone level was evaluated one year after surgery and before only in patients who complained from postoperative erectile dysfunction.

### Cryptorchidism

Until 2008, we had unappropriate data concerning patients with cryptorchidism, but from 2009 on, we had documented informations regarding the initial location of the undescended testes in 83 patients with a history of cryptorchidism in which the overall SRR was 57.8% (48/83).

Twelve patients had at least one intra abdominal or deep inguinal testis at the moment of biopsy but only 4 accepted to undergo surgery with a possibility of orchiectomy and none of them had sperm.

Fifteen patients had at least one palpable inguinal testis. Ten pateints had biopsies with a poor SRR of 10%. The 5 other patients with the same condition underwent orchidopexy 6 months prior to surgery and 1 of them had sperm frozen at biopsy. All bilateral cases had failed biopsies, even if late orchiopexy was performed.

The remaining 64 patients had testes in a scrotal position with a 71.8% SRR (46/64). Nine patients had bilateral orchidopexies with a SRR of 33.3% (3/9) and 55 had unilateral orchidopexies with a 78.2% SRR (43/55) (Table 4).

**Table 4:** Sperm retrieval rate in cryptorchid patients depending on location of the testis at the time of biopsy

Location	n	Orchidopexy	SRR (%)
High impalpable	4	No (4)	0 (0/4)
Palpable inguinal	15	No (*n*=10)	10 (1/10)
		Yes (*n*=5)	20 (1/5)
Scrotal	64	Bilateral (*n*=9)	33.3 (3/9)
		Unilateral (*n*=55)	78.2 (43/55)

*SRR* sperm retrieval rate

In these 46 patients with succeeded biopsies, 36 (75%) had their orchidopexies done before the age of 10 years, regardless of the initial location of the testis.

### Genetic defects

We operated 23 patients with AZFc microdeletions - median age: 36.8 +/− 6.4 - and found sperm in 5 of them (21.7%).

We had 72 Klinefelter patients - median age: 37.8 +/− 4.9 - and 13 of them had sperm at biopsy (18%).

### Testicular tumors

In 11 patients with NOA, preoperative ultrasound revealed infra-clinic testicular tumors. Histopathology revealed benign leydigomas in 6 cases (bilateral in 2 patients), adenoma in one and cancer in 4 patients. Our SRR in such patients was 72.7% (8/11).

## Discussion

Many patients with NOA do have mature spermatozoa within their testes that can be extracted with open biopsies with potential risk of vascular injury and lack of testosterone production [4], all the more since cTESE often removes unnecessary tissue without dilated tubules that are more likely to contain sperm. mTESE as described by Schlegel [15] optimizes the chance of finding spermatozoa with less testicular damage.

### Our technique

The technique we described is a step-by-step macro-mTESE where × 6 loupes are used instead of × 25 operating microscope. Surprisingly, there is poor data on this modified technique in the current literature [16]. Thin and linear tubules are more likely to contain Sertoli-Cell only (SCO) wheras dilated and whitish tubules gathered together with no hyaline connective tissue in between are more likely to contain viable sperm.

As part of the procedure, we perform immediate intraoperative assessment of the retrieved tubules as described by many authors [7, 17]. This helped us to decide if the procedure must go on or terminate [18, 19], so at any step of the procedure, when motile sperm are found, we decide together with the embryologist if sperm retrieval must go on, taking into account the age, the ovarian reserve of the femal partner and the number of oocytes to be retrieved if ICSI is done in a synchronous fashion.

**Fig. 5:**
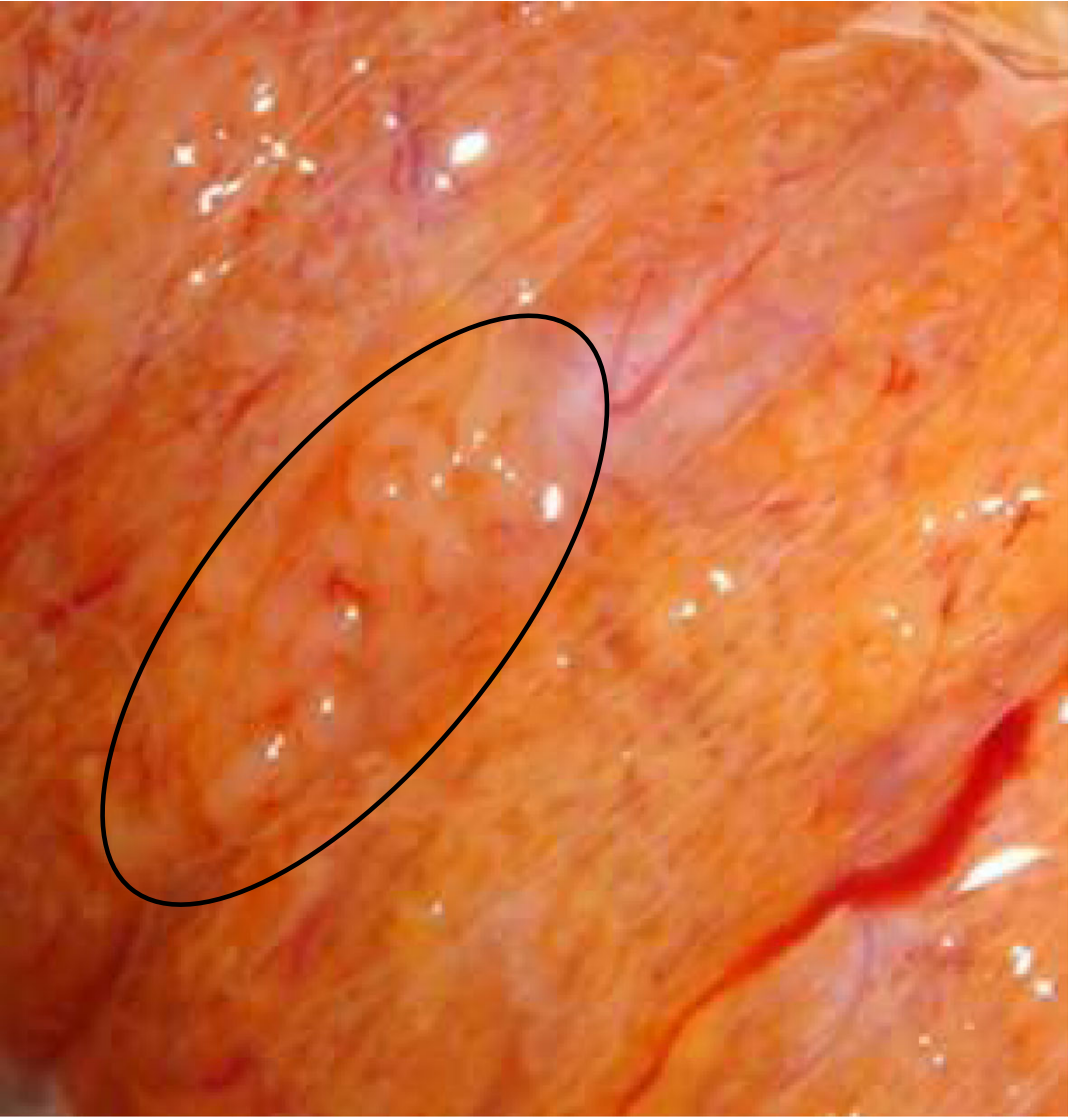
Dilated seminiferous tubules within predominant Sertoli Cell Only pattern

The overall SRR for × 6 loupe-assisted TESE was 51.8% and sperm were found in the first testis in 61% of the patients, leading to unnecessary controlateral procedures. Among these patients, 128 (12.1%) had sperm retrieved far from the superficial mid part of the testis where cTESE is usually done and we assume that without the assistance of × 6 loupes, no sperm would probably have been found in them. Even when dissection demonstrates diffuse SCO, foci of dilated tubules may be found deep in the testis (Fig. 5).

We had 8% of late SRR on the very last biopsies, which demonstrates the wide heterogenecity of the testis in NOA patients. Due to this, we assume that diagnostic biopsy and fine needle aspiration are not good predictive tools in men with NOA [3].

The usual number of testicular biopsies was 15 to 20 from each side. In our institution, the samples are dissected by the embryology team before being examined and we are now planning to pass the dissected samples through a 24G angio catheter and use digestion of the sedimented testicular tissue with collagenase before further analyses if no spermatozoa were seen and we believe that this procedure will enhance our SRR as decribed in the literature [20].

**Fig. 6:**
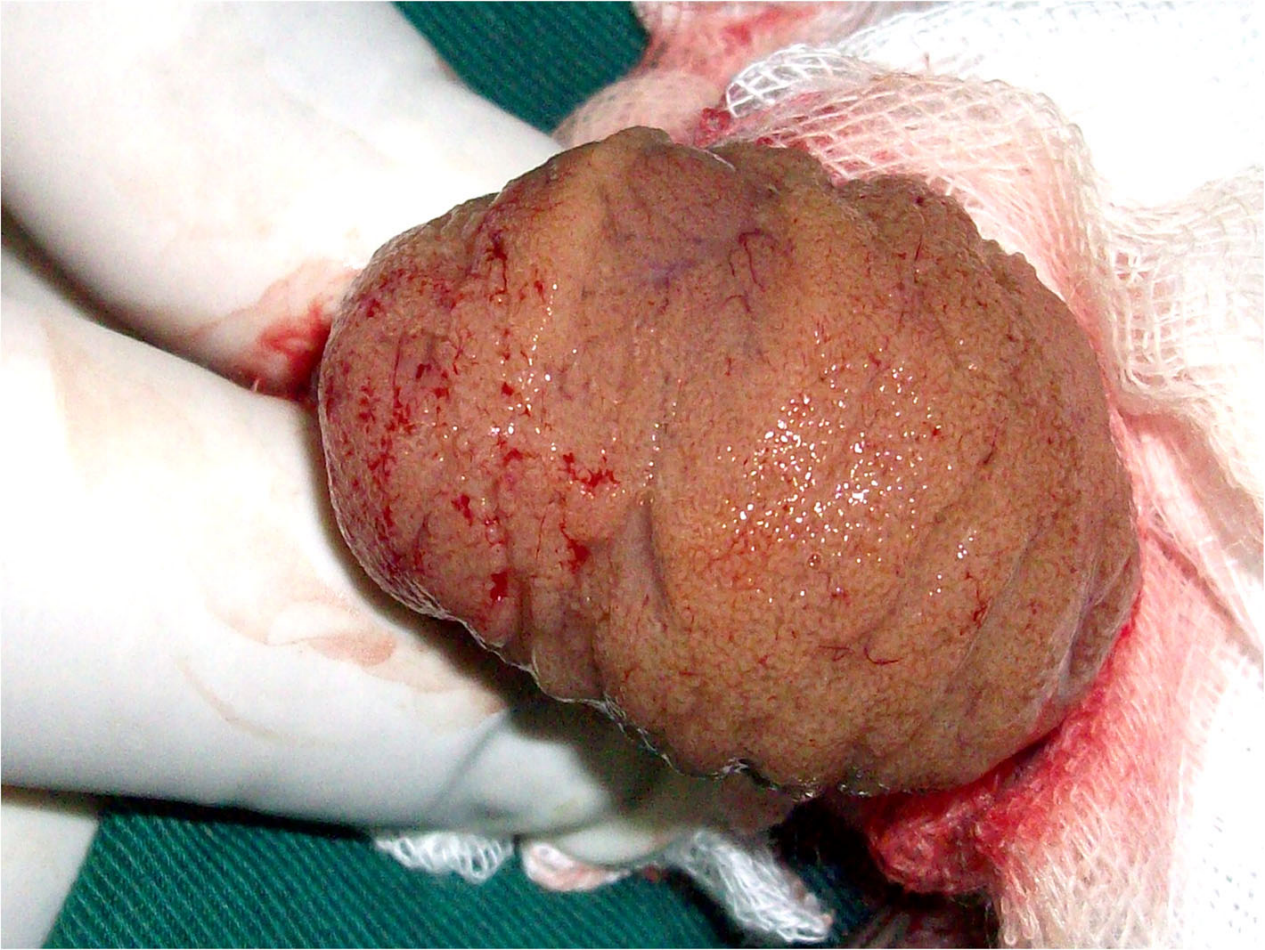
Complete diffuse maturation arrest

In the two last years, despite the fact that we improved our technique with deeper parenchyma dissection and spent less time to identify dilated tubules, we failed to enhance our SRR. This was due to a recruitement bias as many patients referred to us had normal FSH levels and uniform histological pattern of MA with low SRR (Fig. 6) [21]. Incidentally, obstructive azoospermia only accounted 15% of our patients these two last years versus 35% in the early years.

Since we performed × 6 loupe assisted TESE, we have had no testicular atrophy wheras we have had 3 cases with cTESE. Intra testicular haematomas were often diagnosed on ultrasound and they disappeared within 6 months [22, 23]. None had to be evacuated in our series. Deeper dissection was also thought to be more painful until we used local anesthesia.

Since half of our patients came from abroad, we could not assess post operative testosterone levels but this has been evaluated in the literature and testosterone is more likely to reach normal values within one year [24].

In our private clinics in Tunisia, having loupe assistance does not imply any overcost as long as we have a convenient operating time. If we used an operating microscope, the patient would be charged for the extra time that the procedure would demand.

### Predictive factors and correlation with histology

In the literature, SRR with mTESE is correlated with the most advanced pattern of spermatogenesis rather than the predominant pattern of spermatogenesis on the results of the intraoperative testicular assesment [25].

Usually, the sample reffered to histopathology is a random single biopsy of 5x5x5 mm [26] but, in our experience, due to the frequent heterogenocity of the testis, we believe that such a tiny sample cannot always predict the most advanced histological pattern. In addition, when very superficial biopsies allowed to retrieve enough sperm, disection stopped and one could not assess precisely the different histological patterns that are present deeper within the testis. That is why in some cases, we failed to correlate histology to SRR. We are wondering if biopsies from different areas instead of a single one may help to achieve correct histopathology assesment.

We had no data on SRR related to histology but in the literature, patients with early MA have lower SRR than those with late MA. Also, focal MA was associated to higher SRR vs diffuse MA (100% of tubules showing MA) as it has been published [26]. In every histopathological subgroup, patients with heterogeneous tubules seem to have a higher SRR than patients with homogeneous tubules [27].

Many attemps have been made to define predictive models or formulas using non invasive parameters [28–31] but none of them was able to predict unnecessary surgery. Ziaee et al. [32] published that the positive predictive value for a combination of FSH and inhibin B was 100%, this conclusion is made upon a few number of cases (85 patients with 21,2% SRR). FSH, Inhibin levels, testicular volume and negative previous testicular histopathology have failed to predict the presence of spermatozoa within the testes [29, 33–35].

**Fig. 7:**
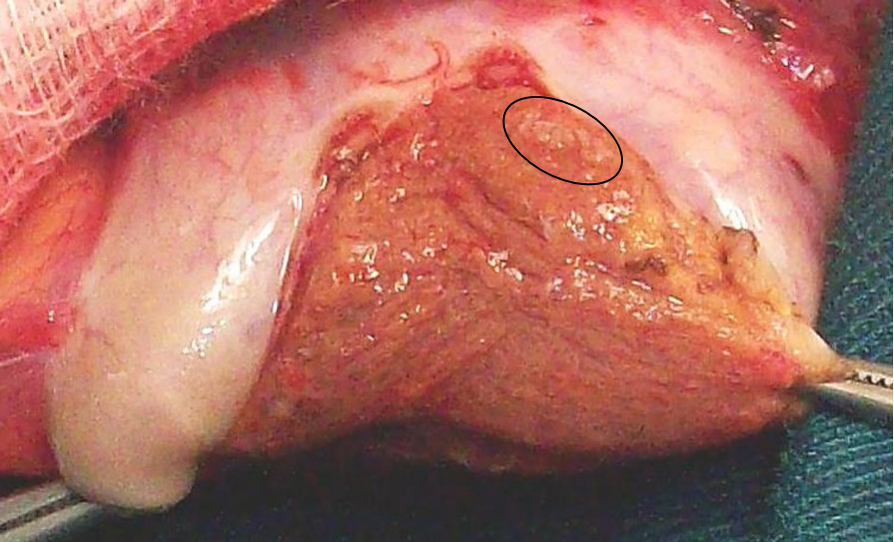
Dilated tubules in the lateral aspect of a 5 ml testis with a high FSH range and predominant Sertoli Cell Only pattern

Smaller testicular volume and high FSH levels are not correlated to a poor SRR for small foci of advanced spermatogenesis may be present in these conditions (Fig. 7) [9, 36]. Patients with testis volume > 10 cc and FSH < 10 are correlated to a poorer SRR [26].

In our series, older age does not adversely affect SRR, which seems to support the idea that elderly patients are more likely to have acquired azoospermia with a higher frequency of hypospermatogenesis than congenital NOA [37].

### Cryptochidism

In our series, the overall SRR was 57.8%. Successfull × 6 Loupe-TESE was correlated to early age of orchiopexy and scrotal location of the testis at the time of biopsy. When the testis was located in a low inguinal position and remained still palpable, we had a poor SRR of 10% which jumped to 20% if the testis could be descended to a scrotal position 6 months prior to biopsy. We never found sperm in patients with unpalpable testes. Compared to other NOA patients, a man with a history of cryptorchidism tends to have a slightly higher chance of sperm retrieval, and even if he had a history of bilateral cryptorchidism, mTESE will lead to find sperm in up to 62% [10, 38].SRR is also correlated to a subnormal testicular volume but independant from FSH [39].

### AZF microdeletions

It is very well documented that men with complete AZFa microdeletion will demonstrate diffuse SCO pattern whereas those with complete AZFb have diffuse MA [40]. None of them will have sperm in their testes and thus, testicular biopsy is contraindicated.

The most frequent microdeletion is AZFc [41]. In this condition, patients may have oligozoospermia, typically < 1 M/ml and freezing, if possible, is mandatory since these patients may continue to impair their spermatogenesis until they become azoospermic [40, 42].

The usual SRR in these AZFc patients is around 55% [42] but in our series, we found sperm in only 21.7% of these patients. All patients with sperm at biopsy had SCO with small foci of spermatogenesis rather than HS. We initially thought that this poor SRR might be due to an older age in this group compared to standard patients but there was no significnt difference. This may only be related to the small number of AZFc patients in this cohort.

### Klinefelter

In klinefelter patients, SRR is strongly correlated to the age of the patient and his testoterone level at the time of surgery [34] although in older patients, SRR is still high if sperm is retrieved with mTESE [43–45].

We had very few cases of Klinefelter in the early years because they were labelled to be infertile. We operated 72 patients and always performed bilateral biopsies since they may continue to impair their spermatogenesis, which would lead to poorer retrieval rates if further biopsies are needed. We had a very poor SRR of 18% and we believe that this is not related to our technique.

This seems to be more likely due to the fact that our patients were not diagnosed as Klinefelters and even had testesterone therapy for a long period, usually for erectile dysfunction, before they were referred to us.

Also, in our country, there is a lack of information on the possibility of sperm retrieval in them [44, 46, 47]. Thus, when they are eventually referred to our centre, they are often over 35 years old, which is correlated to a poor SRR [11]. In our series, the mean age of Klinefelter patients is 37.8 +/− 4.9. We had only one Klinefelter patient that we operated at the age of 23 and we could freeze 3 pellets of viable sperm. ICSI was performed 6 years later, as he got married, which led to one take home baby.

On the other hand, we performed synchronous × 6 loupe TESE in a 41 year-old patient. Surprisingly, not only sperm were found, but the couple could have taken home twins at the first ICSI attempt.

Due to this, we always offer biopsies in Klinefelter patients unless 4 conditions are present: more than 40 years old, previous failed testicular biopsy, testicular volume less than 1,5 ml and low testorene level (< 3 ng/ml) with no response to hormonal stimulation. We usually begin with one tablet every second day of clomifen citrate 50 mg, and if testosterone doen’t increase within one month, we recommand one daily tablet. After a 3 month-period, if teststerone is not beyond 3 ng/ml, we may add 5000 IU of HCG twice a week.

Collaboration between urologists and endocrinologists should be made to discuss wether sperm retrieval should be done by the age of 20, before these young men undergo testosterone replacement therapy, which seems to be a promising option [44].

### Testicular tumors

In 11 patients with NOA, ultrasound revealed unexpected infra-clinic testicular tumors. × 6 loupes were useful to identify the tumor from the surrounding tissue and perform tumorectomy with safe margins, together with TESE. Our SRR in such patients is 72.7%.

In benign cases, we performed conservative surgery and in case of leydigomas, 3 of the 6 patients demonstrated sperm in their ejaculate from 3 to 6 months after surgery. In them, freezing was mandatory because they may have recurrence with de novo NOA.

In a patient with unilateral tumor, when no sperm were found in the ipsilateral testis, we never performed contralateral TESE in the same session.

Conservative treatment may be offered in very few selected patients with testis cancer if 5 conditions are present: tumor size< 20 mm, safety distance from the rete testis, no intraepithelial neoplasia in the remaining parenchyma, normal preoperative testosterone levels, close follow-up [48].

## Conclusion

Physicians are still in need of a reliable marker which will guide them to offer TESE to men with NOA, especially in countries where the whole ICSI procedure is not covered by social security and health insurance. Nevertheless, mTESE is now considered to be the gold standard technique to retrieve sperm in NOA patients. Smaller specimens with dilated tubules are retrieved with less testicular damage and better SRR. Conventional TESE only harvests random samples without any discrimination of dilated foci which are more likely to contain sperm. We described a technique where conventional step-by-step TESE is assisted with × 6 magnifying loupes together with deep microdissection of the testes and intra operative assesment of the samples. In institutions with lack of enough operating theaters where long procedures can take place, or when operating microscope is not available, the use of × 6 loupes will permit to enhance SRR in an acceptable operating time compared to cTESE, regardless of the aetiology of NOA.

## Data Availability

The datasets used and/or analyzed during the current study are available from the corresponding author on reasonable request.

## Abbreviations

AZF: Azoospermic factor
CTESE: Conventional TESE
FNA: Fine needle aspiration
FSH: Follicule stimulating hormone
HCG: Human chorionic gonadotrophin
HS: Hypospermatogenesis
ICSI: Intratesticular sperm injection
IVF: In vitro fertilisation
MA: Maturation arrest
mTESE: Micro testicular sperm extraction
NOA: Non obstructive azoospermia
SCO: Sertoli cell only
SRR: Sperm retrieval rate
ST: Seminiferous tubules
